# Echocardiographic image quality deteriorates with the severity of cardiogenic shock

**DOI:** 10.1186/s43044-024-00544-9

**Published:** 2024-08-23

**Authors:** Hazem Lashin, Francesco Vasques, Sanjeev Bhattacharyya

**Affiliations:** 1 Adult Critical Care Unit, Barts Heart Centre, St Bartholomew’s Hospital, West Smithfield, London, EC1A 7BE UK; 2 William Harvey Research Institute, Barts and the London School of Medicine and Dentistry, Queen Mary University of London, Charterhouse Square, London, UK; 3 Adult Critical Care Unit, St Thomas’s Hospital, London, UK; 4 Cardiology Department, Barts Heart Centre, St Bartholomew’s Hospital, West Smithfield, London, UK

**Keywords:** Cardiogenic shock, Echocardiography, Image quality, Myocardial infarction

## Abstract

**Background:**

Transthoracic echocardiography (TTE) is the primary tool for assessing left ventricular (LV) function in cardiogenic shock (CS). However, inadequate image quality often hinders it. In this retrospective study, we investigated factors associated with LV image quality in patients admitted to the intensive care unit (ICU) with ischemic CS.

**Results:**

Two critical care physicians accredited in echocardiography independently reviewed the TTEs of 100 patients admitted to our tertiary cardiac ICU with ST-elevation myocardial infarction complicated by CS between October 2016 and September 2019. Endocardial border definition (EBD) was graded for each myocardial segment of the apical 4-chamber and 2-chamber views using a conventional scoring system (1 = good, 2 = suboptimal, 3 = poor, and 4 = not possible). The biplane EBD index (EBDi) was calculated by averaging all segments from both views. An average EBDi of both observers was correlated with clinical and echocardiographic parameters. The median age was 62 years [54, 73], and 78% were males. LV ejection fraction and cardiac index (CI) medians were 29% [20, 35] and 1.93 l/min/m^2^ [1.40, 2.51], respectively. The median biplane EBDi was nearly suboptimal (1.833 [1.542, 2.083]). There was no correlation between EBDi and age, sex, or body mass index. However, biplane EBDi demonstrated statistically significant correlations with PaO_2_ (*r*^2^ = 0.066, *p* = 0.01), mean arterial pressure (MAP, *r*^2^ = 0.055, *p* = 0.03), CI (*r*^2^ = 0.105, *p* < 0.01), tricuspid annulus systolic velocity (RV S’, *r*^2^ = 0.092, *p* = 0.01), and tricuspid regurge maximum velocity (TR Vmax, *r*^2^ = 0.067, *p* = 0.01). In a multivariate model, only CI correlated independently with EBDi (*r*^2^ = 0.105, *p* < 0.01). The biplane EBDi predicted CI (area under the curve (AUC) 0.70, *p* = 0.001) with good sensitivity (71%) and reasonable specificity (61%).

**Conclusions:**

The study suggests that in patients admitted to the ICU with ischemic CS, LV image quality by TTE deteriorates with the severity of shock, as indicated by CI.

**Supplementary Information:**

The online version contains supplementary material available at 10.1186/s43044-024-00544-9.

## Background

One in ten ST-elevation myocardial infarctions (STEMI) is complicated by cardiogenic shock (CS) [1]. Patients with ischemic CS often require admission to the intensive care unit (ICU) and suffer high mortality, up to 40% [2, 3]. Effective management of CS requires timely diagnosis, careful monitoring, and prompt treatment.

The use of transthoracic echocardiography (TTE) is essential for managing critically ill patients with ischemic CS. TTE can diagnose and identify the severity of left ventricular (LV) dysfunction. When combined with other forms of hemodynamic monitoring (such as pulmonary artery catheter), TTE becomes a crucial tool in managing CS. TTE enables the healthcare provider to repeatedly assess cardiac function, detect complications (e.g., ventricular septal defect), monitor hemodynamics, and evaluate the response to treatment over time.

The left ventricle ejection fraction (LVEF) by modified Simpson’s method of disks is the most common technique for assessing LV function [4]. This method estimates the LV volumes by tracing the end-diastolic and end-systolic endocardium in two orthogonal echocardiographic views (apical four-(A4C) and two-chamber (A2C)) [5]. Accordingly, accurate LVEF relies heavily on endocardial border definition and image quality, often compromised in critically ill patients. In a previous study, 74% of ICU patients with ischemic CS received only a visual estimate of LVEF because of insufficient endocardial border definition. In comparison, neither Simpson’s nor visual LVEF assessment was possible in 16% of the cases due to poor acoustic windows [6, 7].

This problem is frequently encountered in the ICU [7]. In patients who underwent cardiac surgery, TTE image quality was compromised by age, male sex, and temporal proximity to surgery [8]. In the non-critically ill patients, age, male sex, and obesity were the main compromising factors [9]. To our knowledge, no previous published attempt has been made to identify the parameters associated with poor echocardiographic image quality in CS. This neglected area of echocardiography research is critical for improving the diagnostic yield of such an increasingly utilized imaging modality. Advances in this field are especially important in CS, where echocardiography is a cornerstone of diagnosis and management.

We hypothesized that age, male sex, and body habitus are associated with LV echocardiographic image quality in patients admitted to ICU with ischemic CS and reduced LVEF (< 40%). This retrospective study investigated the association between age, sex, body surface area (BSA), and body mass index (BMI) with LV endocardial border definition (EBD). Additionally, we explored other clinical and echocardiographic factors that correlate with EBD in the same cohort.

## Method

### Patients

We retrospectively reviewed consecutive patients admitted with a diagnosis of STEMI complicated by CS to the ICU of our tertiary cardiac center between October 2016 and September 2019. The inclusion criteria were age > 18 years, STEMI followed by primary percutaneous coronary intervention (PCI) with CS requiring ICU admission, impaired LV systolic function, and echocardiography within 48 h of ICU admission. CS was defined as a systolic arterial pressure less than 90 mmHg or a mean arterial pressure less than 65 mmHg, with or without therapy, and evidence of organ hypoperfusion (e.g., delirium, acute kidney injury, acute liver injury) [10, 11]. LV systolic impairment was defined as LVEF less than 40% [12]. The exclusion criteria were age less than 18 years, pregnancy, non-cardiogenic cause of shock, cardiac arrest with early signs of cerebral hypoxia, or patients considered moribund by the admitting physician (defined as imminent death with no medical therapeutic option). We excluded patients who could not have their LV assessed by TTE. This was necessary to adhere to the definition of CS with impaired LVEF and to investigate the correlation between echocardiographic parameters and image quality. Patients excluded due to poor image quality received other modalities for clinical cardiac assessment, including point-of-care ultrasound, TTE enhanced with ultrasound-enhancing agents or transesophageal echocardiography. The study was conducted in accordance with the Declaration of Helsinki. The study was approved by the Institutional Review Board (IRB), and the IRB waived the requirement for written informed consent.

### Data

We collected patients’ demographics, comorbidities, medical history, clinical parameters, laboratory tests, medications, APACHE II severity score, presence of mechanical ventilation, mechanical circulatory support (MCS) and renal replacement therapy (RRT), length of stay, and 28-day mortality by reviewing electronic case records. Clinical parameters were recorded as the worst documented values on day one of ICU admission. The APACHE II score was calculated as previously described [13].

### Echocardiography

Echocardiography was performed by experienced accredited (British Society of Echocardiography (BSE) level 2 or equivalent) cardiac sonographers at the bedside in the ICU using commercially available ultrasound systems with a 3.5-MHz probe (GE Health Care, Horten, Norway). A dedicated electronic central storage facility was used for the images and reports. Echocardiograms, including chamber quantification, were obtained according to the BSE recommendations [14]. Chamber quantification data were collected from stored images and reports by the study team, including LVEF by Simpson’s biplane or visual estimate at the time of the original scan, LV outflow tract (LVOT) velocity time integral (VTI) by pulsed wave Doppler at 0.5 to 1 cm proximal to the aortic valve, LVOT diameter, right ventricular (RV) dimensions, right ventricle outflow tract (RVOT) dimensions, RVOT VTI by pulsed wave Doppler at 0.5 to 1 cm proximal to the pulmonary valve, tricuspid annular plane systolic excursion (TAPSE) by M-mode of the lateral tricuspid annulus and Tricuspid annulus peak systolic velocity (RV S’) by tissue Doppler at the lateral tricuspid annulus [14]. Tricuspid regurgitation maximum velocity (TR Vmax) was investigated using continuous-wave Doppler through the tricuspid valve during systole. SV, cardiac output (CO), and cardiac index (CI) were calculated as follows: LVOT VTI x π x LVOT radius^2^, SV x heart rate, and CO/body surface area (BSA), respectively. The left atrial (LA) diameter was measured at end-systole, parallel to the mitral annulus. The LA and right atrial areas were obtained by tracing the LA and RA in the apical-4-chamber view at end-systole. Mitral E, A, and E deceleration time (DecT) waves were measured using pulsed-wave Doppler at the mitral valve leaflet tips during diastole. Furthermore, tissue Doppler echocardiography was used to measure septal and lateral e’ velocities. The E/e’ ratio was calculated as the E velocity/average e’ (septal and lateral). The inferior vena cava (IVC) diameter was measured as the largest at 1 cm distal to the right atrium.

### Assessment of echocardiographic image quality

We assessed the echocardiographic image quality of the conventional apical 4-chamber (A4C) and apical 2-chamber (A2C) views required for LVEF calculation. All echocardiograms were reviewed retrospectively and independently by two critical care physicians accredited (BSE–TTE level 2) and experienced in echocardiography. Each view was divided into six segments described in the BSE guidelines [15]. EBD was graded using a conventional scoring system [16, 17]. A score of 1 indicated good endocardial border definition, 2 indicated suboptimal (the endocardial border is only partially visualized), and 3 indicated poor definition (endocardial border not visualized). If a segment was not visible, it received a score of 4. A mean of the two independent observers was used as the EBD score. To determine the EBD index (EBDi), the scores for all segments in a specific view are added together and then divided by the number of segments in that view. For the biplane EBDi, the mean of the scores from both views is calculated.

### Endpoints

The primary endpoint was the correlation between age, male sex, and two surrogates for body habitus (body surface area (BSA) and body mass index (BMI)) with the LV’s biplane EBDi in this cohort. The secondary endpoint was the correlation between other clinical and echocardiographic markers with the biplane EBDi in the same cohort.

### Statistical analysis

Categorical data are expressed as numbers and percentages. Medians with interquartile ranges [IQR] were used to summarize continuous variables according to their distribution. Nonparametric continuous variables were compared using the Mann–Whitney U test. Linear and logistic regressions were used to investigate the correlations between continuous variables and other continuous and categorical variables, respectively. The univariate linear and logistic regression results are expressed as *r*^2^ and odds ratio (OD) with a 95% confidence interval (CI), respectively. Receiver operating characteristic (ROC) curve analysis was used to test the sensitivity of the logistic regression model. ROC analysis results were expressed as the area under the curve (AUC) and provided values with the best sensitivity and specificity for the regression model. Variables that correlated significantly with biplane EBDi in univariate analysis were used in a multivariate regression model to determine the independent variables that could predict the biplane EBDi. Pearson’s coefficient and the Bland–Altman test assessed the agreement and differences between observers in 10 views selected using a random number generator. Statistical significance was set at *p* < 0.05. A statistical analysis plan was defined before the analysis, and missing data were not imputed. The Prism GraphPad version 10 software package for macOS was used for statistical analysis.

## Results

**Fig. 1:**
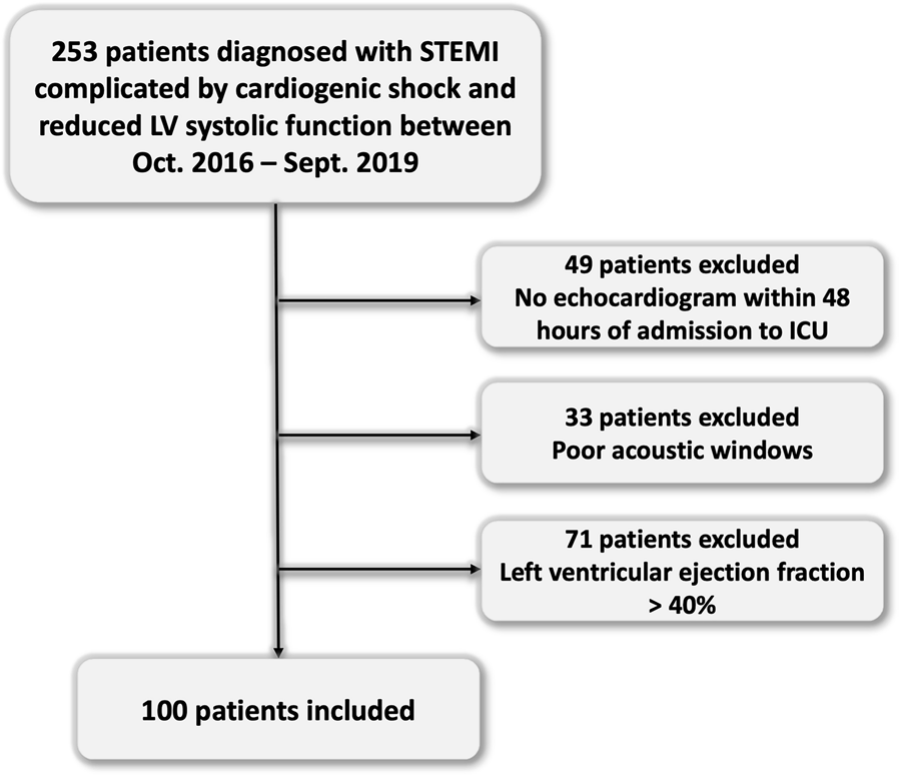
Inclusion and exclusion chart. ICU: intensive care unit. LV: Left ventricle. STEMI: ST-elevation myocardial infarction

During the study period, 253 consecutive patients with a diagnosis of STEMI complicated by CS were admitted to the ICU. Forty-nine patients did not undergo comprehensive echocardiography within 48 h of admission to the ICU, 33 had poor windows prohibiting LVEF assessment at the time of the original scan, and 71 had LVEF > 40% and were excluded (Fig. 1). Therefore, 100 patients were included in this study [3]. The participants’ median age was 62 years [54, 73], 78% were male, the median APACHE II score was 17 (13–21), and the mortality was 37% at 28 days (Table 1). The median BSA and BMI were 1.9 m^2^ [1.8, 2.1] and 26.5 kg/m^2^ [24.2, 29.9], respectively.

**Table 1:** Demographics and clinical parameters

Number of patients	100
Age, years [IQR]	62 [54, 73]
Gender, No (%)	78 males (78%), 22 females (22%)
Body surface area, m^2^ [IQR]	1.9 [1.8, 2.1]
Body mass index, kg/m^2^ [IQR]	26.5 [24.2, 29.9]
Out of hospital cardiac arrest, No (%)	38 (38%)
APACHE II score, median [IQR]	17 [7, 13]
28-day mortality, No (%)	37 (37%)
Mechanical ventilation, No (%)	85 (85%)
Mechanical circulatory support, No (%)	40 (40%)
Renal replacement therapy, No (%)	25 (25%)
Intensive care unit length of stay, median [IQR]	6 [4, 11]
Hospital length of stay, median [IQR]	17 [8, 25]
Clinical parameters	
pO_2_, kPa [IQR]	9.4 [8.6, 10.4]
FiO_2_ [IQR]	0.30 [0.25, 0.45]
pO_2_/FiO_2_ ratio (SD)	30.33 (12.71)
Respiratory rate, breaths per minute [IQR]	24 [20, 28]
pH, (SD)	7.25 [7.19, 7.32]
Heart rate, beats per minute [IQR]	84 [68, 96]
Mean arterial pressure, mmHg [IQR]	68 [61, 91]
Lactate, mmol/l [IQR]	3.3 [2.3, 6.6]
Urea, mmol/l [IQR]	8.2 [6.4, 11.8]
Creatinine, mmol/l [IQR]	121 [98, 157]
Bilirubin, mmol/l [IQR]	10 [6, 14]
Medical history	
Previous CABG, No (%)	4 (4%)
Heart Failure, No (%)	7 (7%)
Asthma, No (%)	9 (9%)
Chronic obstructive airway disease, No (%)	3 (3%)

APACHE II: Acute Physiology and Chronic Health Evaluation II, CABG: coronary artery bypass grafting, IQR: Interquartile range, No: number, SD: Standard deviation

The review of echocardiography revealed a median LVEF of 29% [20, 35] and CI of 1.93 l/min/m^2^ [1.40, 2.51]. Also, TAPSE, RV S’, RVOT VTI, and TR Vmax medians [IQR] were 1.6 cm [1.2, 1.9], 11 cm/s [8, 14], 12.2 cm [10.6, 17.0], and 2.0 m/s [1.4, 2.5], respectively. (Table 2) The semi-quantitative review of the echocardiographic image quality revealed the median [IQR] EBDi of the A4C view, A2C views, and biplane to be 1.708 [1.438, 1.979], 1.917 [1.583, 2.250], and 1.833 [1.542, 2.083], respectively. The two observers correlated strongly in the EBDi assessment (*r* = 0.76, *p* < 0.01); however, one observer consistently assigned higher EBD scores with a mean bias of 0.200.

**Table 2:** Echocardiographic parameters

Echocardiography parameter	Number	Result
Right ventricle		
TAPSE, cm [IQR]	97	1.6 [1.2, 1.9]
RV S’, cm/s [IQR]	69	11 [8, 14]
TR Vmax, m/s (IQR)	92	2.0 [1.4, 2.5]
RVOT VTI, cm (IQR)	42	12.2 [10.6, 17.0]
RV basal diameter, cm [IQR]	94	3.8 [3.3, 4.2]
Left ventricle		
EF, % [IQR]	99	29 [20, 35]
Cardiac Index, l/min/m^2^ [IQR]	92	1930 [1400, 2510]
E velocity, m/s [IQR]	92	0.84 [0.56, 0.99]
A velocity, m/s [IQR]	91	0.58 [0.40, 0.81]
E/A ratio [IQR]	91	1.2 [0.86, 1.82]
DecT, ms [IQR]	92	160 [126, 204]
Average e’, m/s [IQR]	79	0.07 [0.05, 0.08]
E/e’ ratio [IQR]	79	12.8 [8.9, 15.6]
Right atrium		
Area, cm^2^ [IQR]	93	15.4 [12.5, 20.2]
Left atrium		
Diameter, cm [IQR]	91	3.7 [3.2, 4.2]
Area, cm^2^ [IQR]	95	20.8 [15.8, 24.8]
Inferior vena cava		
Diameter, cm [IQR]	61	2.3 [2.0, 2.6]

DecT**:** E velocity deceleration time, EF: Ejection fraction, IQR: Interquartile range, LVOT: Left ventricle outflow tract, RV: Right ventricle. RV S’: Tricuspid annulus peak systolic velocity, RVOT: Right ventricular outflow tract, TAPSE: Tricuspid annular plane systolic excursion, TR Vmax: Tricuspid regurgitation maximum velocity, VTI: Velocity time integral

**Fig. 2:**
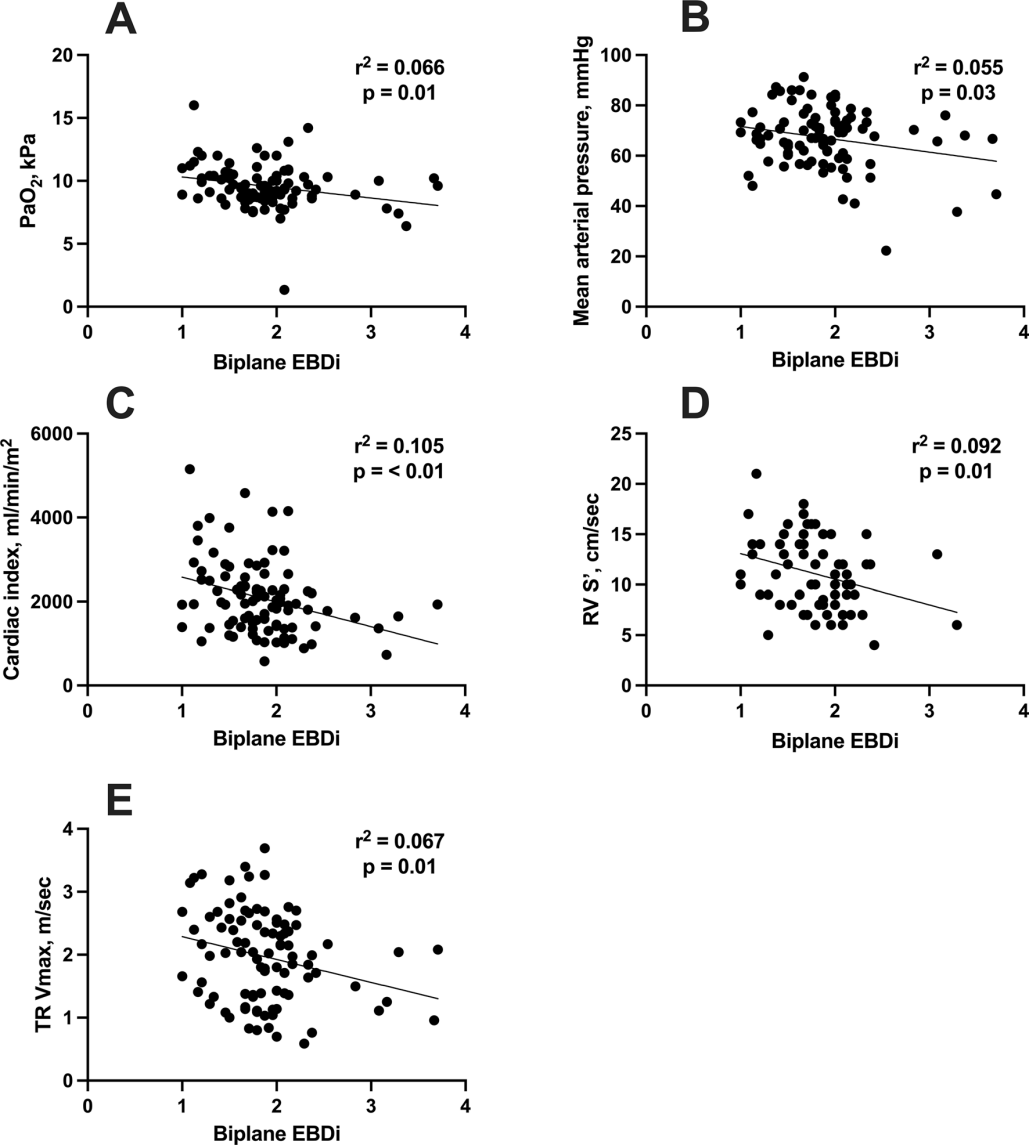
Correlation between clinical and echocardiographic parameters and biplane EBDi. **A** PaO2, **B** Mean arterial pressure, **C** Cardiac index, **D** RV S’, **E** TR Vmax. CI: Cardiac index. EBDi: Endocardial border definition index. RV S’: Tricuspid annulus plane systolic velocity. TR Vmax: Tricuspid regurgitation maximum velocity

Univariate regression analysis investigated the correlation between age, sex, BSA, BMI, and the biplane EBDi in this cohort. The analysis revealed that there was no statistically significant correlation between age (*r*^2^ = 0.001, *p* = 0.76), sex (OD 0.86 confidence interval 0.36, 2.18, *p* = 0.11), BSA (*r*^2^ = 0.013, *p* = 0.26), or BMI (*r*^2^ = 0.033, *p* = 0.14) with the biplane EBDi. Supplementary Table 1 provides additional details on these findings. However, biplane EBDi demonstrated statistically significant correlations with PaO_2_ (*r*^2^ = 0.066, *p* = 0.01), MAP (*r*^2^ = 0.055, *p* = 0.03), CI (*r*^2^ = 0.105, *p* < 0.01), RV S’ (*r*^2^ = 0.092, *p* = 0.01), and TR Vmax (*r*^2^ = 0.067, *p* = 0.01) (Supplementary Table 1, Fig. 2). LA diameter exhibited a trend toward association with biplane EBDi (*r*^2^ = 0.040, *p* = 0.06) but did not reach statistical significance. A multivariate regression model was constructed to include all statistically significant univariate variables to investigate the independent predictors of biplane EBDi. The model revealed that CI (− 0.001 (− 0.001 to − 3.366), *p* = 0.04) was an independent predictor of biplane EDBi. There was no significant collinearity between the variables (Supplementary Fig. 1).

**Fig. 3:**
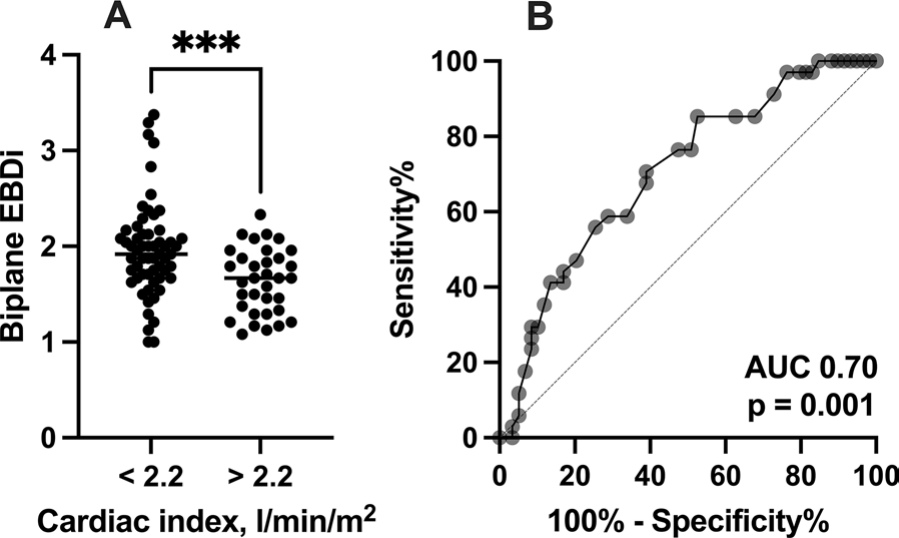
Biplane EBDi based on the cardiac index and TR Vmax clinical cutoffs. **A** Biplane EBDi comparison between patients with a cardiac index above and below 2.2 l/min/m^2^. **B** ROC curve of biplane EBDi predicting cardiac index above and below 2.2 l/min/m^2^

**Fig. 4:**
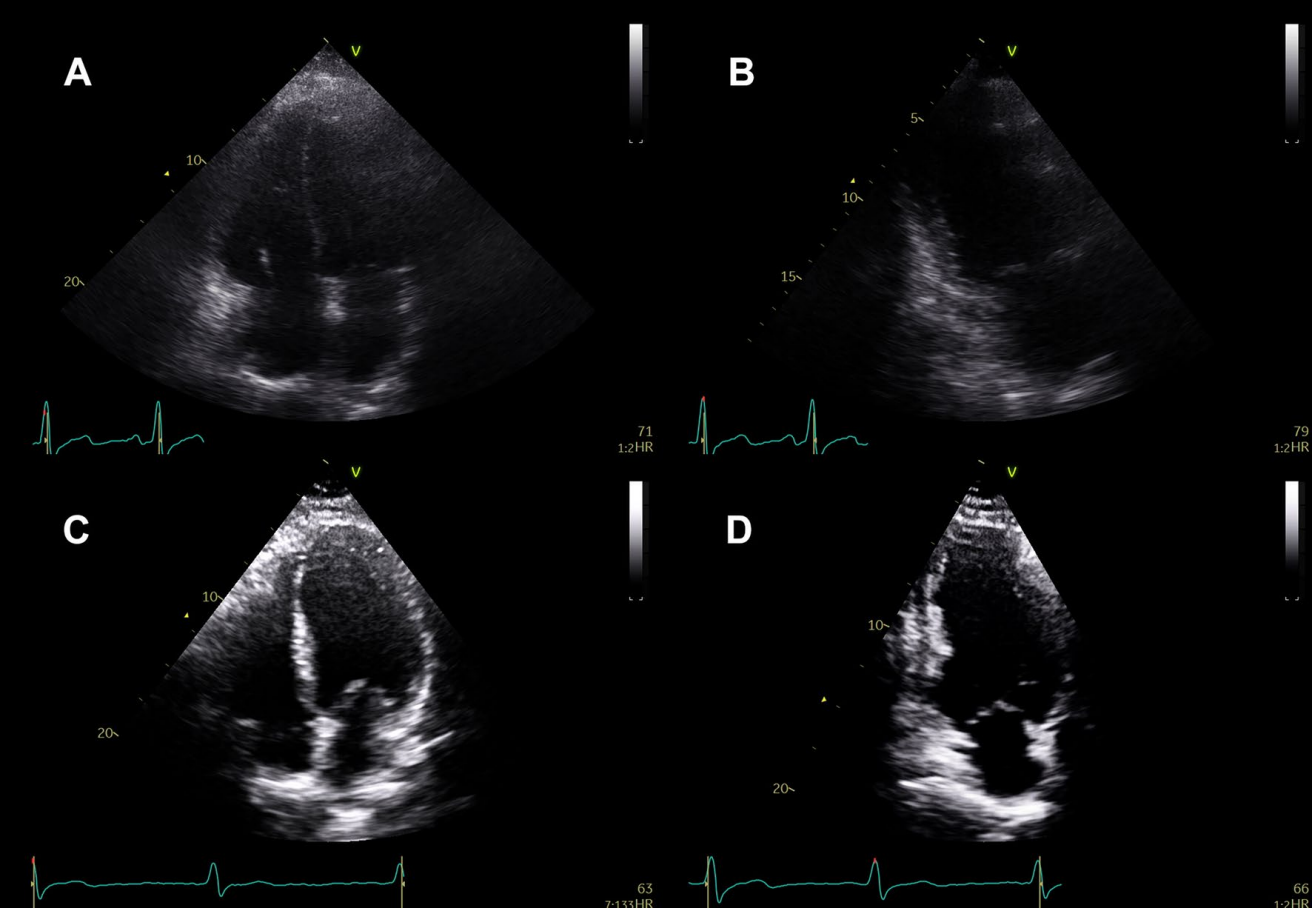
Apical-4- and apical-2-chamber views of patients with a cardiac index below and above 2.2 l/min/m^2^. **A** Apical-4-chamber view of a patient with a 1.4 l/min/m^2^ cardiac index. **B** Apical-2-chamber view of a patient with a 1.4 l/min/m^2^ cardiac index. **C** Apical-4-chamber view of a patient with a 2.9 l/min/m^2^ cardiac index. **D** Apical-2-chamber view of a patient with a 2.9 l/min/m^2^ cardiac index

Patients were then categorized based on the CI cutoff for CS (2.2 l/min/m^2^). Patients (*n* = 59) with a CI < 2.2 l/min/m^2^ exhibited significantly higher (worse) biplane EBDi than patients (*n* = 34) above the cutoff (1.917 vs. 1.667, *p* = 0.0009, Fig. 3A). ROC analysis revealed that the biplane EBDi demonstrated a good AUC (0.70, *p* = 0.001) for predicting CI above or below 2.2 l/min/m^2^ (Fig. 3B). An EBDi < 1.854 predicted CI with good sensitivity (71%) and reasonable specificity (61%). Figure 4 and Videos 1–4 demonstrate examples of A4C and A2C views in patients with CI below and above 2.2 l/min/m^2^.

## Discussion

In this retrospective single-center study, we observed that the LV’s echocardiographic image quality was close to the suboptimal threshold in patients receiving intensive care for ischemic CS. In line with anecdotal evidence, the EBDi in the A2C view was worse than in the A4C view. We investigated the potential contribution of many clinical and echocardiographic factors to the suboptimal EBDi in this cohort. Contrary to previous studies and our hypothesis, age, sex, and body habitus did not correlate with image quality in this cohort. However, oxygenation, blood pressure, RV systolic function, and CI were associated with EBDi. Further analysis revealed that EBDi was significantly worse in patients with worse CI. Furthermore, the CI was an independent predictor of biplane EBDi in this cohort. These findings indicate that the LV echocardiographic image quality deteriorated as shock worsened in patients admitted to ICU with ischemic CS.

Ischemic CS is characterized by depressed myocardial contractility secondary to an inadequate coronary blood supply, resulting in systolic and diastolic dysfunction. The systolic component results in low cardiac output, leading to hypotension and poor organ perfusion. Poor contractility results in diastolic failure and increased LVEDP, leading to high left atrial pressure and pulmonary edema. Our results suggest that systolic and diastolic cardiac failure markers were associated with poor echocardiographic image quality in Ischemic CS. Our cohort's suppressed systolic parameters (CI and MAP) were associated with a worse EBDi. This systolic dysfunction is associated with microvascular failure and mitochondrial dysfunction, resulting in localized edema [18]. This process applies to the chest wall tissues, rendering ultrasound penetration more difficult and degrading echocardiographic image quality. Furthermore, RV systolic dysfunction (RV S’) was associated with reduced image quality in this cohort. This reduction in EBDi may be due to systemic venous congestion, leading to tissue edema and further barriers to ultrasound. Similarly, worse PaO_2_ and LA diameter (trending toward statistical significance) were also associated with difficult LV imaging. These surrogates for pulmonary edema and LA pressure indicate that poor diastolic function leads to increased lung water and reduced ultrasound reach. The CI exhibited the strongest correlation with EBDi and was an independent predictor in this cohort, suggesting that CS's underlying physiological mechanism is the driving force behind these observations. Furthermore, we observed that patients with impaired oxygenation, low blood pressure, and CI exhibited worse LV image quality by echocardiography. This may be attributed to the hesitancy of the echocardiographer in modifying the patient's position due to their fragile condition, which may ultimately result in a further decline in the quality of the image.

Body habitus has been linked to the quality of echocardiographic images, particularly in relation to increased BMI. In a previous study, increased BMI was associated with worse point-of-care LV echocardiographic image quality in a cohort of patients presenting to the emergency department [19]. The median BMI for this cohort was 35.6 kg/m^2^, and the LV endocardial definition deteriorated significantly in those with BMI > 30 kg/m^2^. Furthermore, another study of more than 1000 non-critically ill patients with a median BMI of 28 kg/m^2^ demonstrated that BMI > 30 kg/m^2^ was associated with nondiagnostic TTEs [9]. Additionally, obesity was associated with increased use of ultrasound-enhancing agents (UEA) to improve image quality. In contrast, our cohort’s median BMI (26.5 kg/m^2^) was lower compared to the previous two, and contrary to our hypothesis, higher BMI was not associated with worse image quality. The lower BMI in our cohort may partly account for why body habitus did not significantly contribute to image quality in our current study.

Male sex and older age were also previously associated with worse image quality in echocardiography. In a study of non-critically ill patients, the male sex represented 49% of the population and was independently associated with nondiagnostic TTE scans [9]. In the same study, older age (> 65) was associated with higher use of UEA, indicating worse image quality. Similarly, in a study examining TTE image quality following cardiac surgery, male sex, and increasing age were both independently associated with worse LV EBD [8]. However, in the current cohort, no significant associations were found between sex or age and image quality. This discrepancy raises questions about the potential differential impact of disease processes and chronicity of illness on the factors influencing echocardiography image quality, warranting further investigation.

The current study observed that LV image quality worsens as disease severity expressed as CI worsens. This cohort of patients with worse CS would benefit most from UEA. However, there is a reluctance to use UEA in severely ill patients, thus denying better diagnosis and guidance of management to those who need it most. This hesitancy remains despite the UEA being proven safe and effective in critical care, including in the most challenging patients [7, 20–22]. Furthermore, we have recently demonstrated in a different study that UEA is effective and safe in the sickest patients, as it is supported by extracorporeal membrane oxygenation (ECMO) [17]. Also, guidelines advocate using UEA in patients in critical care where two LV segments are inadequately visualized [23, 24]. Additionally, recently published guidelines specifically mention that UEA use should not be denied based on any diagnosis in the ICU [25]. Moreover, UEA use with echocardiography was associated with improved diagnostic yield, reduced cost, and a positive impact on patient management in the ICU [26]. This study raises awareness of the worse image quality in patients with severe illness and is a step toward routine application of UEA in this cohort and the wider ICU.

Echocardiography image quality is a problem in intensive care. The current study confirms that the echocardiographic image quality is compromised in CS patients. Furthermore, it confirms anecdotes that patients with worse CS have worse image quality. In a clinical setting, these findings may be helpful for the echocardiography practitioner to optimize image quality as much as possible in such patients. Moreover, it may encourage the use of other echocardiography modalities (transesophageal) or ultrasound enhancing agents (UEA) at the severe end of the CS spectrum. Additionally, these results may trigger further research in this neglected area to improve image quality in CS and develop technology more suitable for this difficult-to-image cohort. The discrepancy between our results and the limited published data indicates that factors influencing image quality may vary according to the disease process, which must be considered when planning research in this field. Also, this research would aid the development of additional studies investigating patients with CS where image quality prevented LV assessment (excluded from the current study and represents a different phenotype), considering patients’ hemodynamics and disease severity along with other factors.

### Limitations of the study

It is important to note that the current study has limitations due to its single-center retrospective design, which may affect its generalizability. Additionally, the study's semi-quantitative nature introduces subjectivity that could lead to bias. It is also worth noting that patients with poor echocardiographic image quality were not included to adhere to the definition of CS with reduced LVEF (< 40%) and to facilitate investigating echocardiographic factors associated with image quality. While mechanical ventilation was not found to impact image quality in this cohort, it is worth noting that ventilator pressures were not included in the analysis. Finally, a larger sample size could have revealed additional independent factors associated with LV image quality.

## Conclusion

In conclusion, our study highlights the suboptimal LV echocardiographic image quality in patients with ischemic CS admitted to ICU. Contrary to the literature and our hypothesis, age, gender, and body habitus were not significantly associated with image quality in this cohort. However, CI was independently associated with the biplane EBDi, indicating that LV image quality deteriorated as shock worsened. The results of our study emphasize the need for further research to investigate the correlation between hemodynamic parameters and echocardiographic image quality in patients with CS, which could lead to improved diagnostic yield and better management.

### Supplementary Information

Supplementary Material 1.

Supplementary Material 2: Video 1. Apical-4-chamber view of a patient with a 1.4 l/min/m^2^ cardiac index.

Supplementary Material 3: Video 2. Apical-2-chamber view of a patient with a 1.4 l/min/m^2^ cardiac index.

Supplementary Material 4: Video 3. Apical-4-chamber view of a patient with a 2.9 l/min/m^2^ cardiac index.

Supplementary Material 5: Video 4. Apical-2-chamber view of a patient with a 2.9 l/min/m^2^ cardiac index.

## Abbreviations

A2C: Apical-2-chamber view
A4C: Apical-4-chamber view
APACHE: Acute Physiology and Chronic Health Evaluation score
AUC: Area under the curve
BMI: Body mass index
BSA: Body surface area
BSE: British Society of Echocardiography
CI: Cardiac index
CO: Cardiac output
CS: Cardiogenic shock
DecT: Mitral E wave deceleration time
EBD: Endocardial border definition
EBDi: Endocardial border definition index
ICU: Intensive care unit
IQR: Interquartile range
IRB: Institutional Review Board
IVC: Inferior vena cava
LA: Left atrium
LV: Left ventricle
LVEF: Left ventricular ejection fraction
LVOT: Left ventricular outflow tract
MCS: Mechanical circulatory support
OD: Odds ratio
PCI: Percutaneous coronary intervention
RA: Right atrium
RRT: Renal replacement therapy
ROC: Receiver operator curve
RV: Right ventricle
RV S’: Tricuspid annulus peak systolic velocity
RVOT: Right ventricular outflow tract
STEMI: ST-elevation myocardial infarction
SV: Stroke volume
TAPSE: Tricuspid annular plane systolic excursion
TR Vmax: Tricuspid regurgitation maximum velocity
TTE: Transthoracic echocardiography
UEA: Ultrasound enhancing agent
VTI: Velocity time integral
