# Supplementary material for: Echocardiographic image quality deteriorates with the severity of cardiogenic shock
Source: Egypt Heart J. 2024 Aug 23;76:110. doi: 10.1186/s43044-024-00544-9 (PMC11343954; doi:10.1186/s43044-024-00544-9)
Supplement: Supplementary file 1 — Supplementary Material 1. [file 43044_2024_544_MOESM1_ESM.docx]

|  | Univariate |  | Multivariate |  |
| --- | --- | --- | --- | --- |
| Parameter | r^2^ or OD (confidence interval) | p value | OD (confidence interval) | p value |
| Age | 0.001 | 0.76 |  |  |
| Gender | 0.86 (0.36, 2.18) | 0.11 |  |  |
| Outcome | 0.53 (0.23, 1.16) | 0.12 |  |  |
| OHCA | 0.71 (0.31, 1.56) | 0.41 |  |  |
| BSA | 0.013 | 0.26 |  |  |
| BMI | 0.023 | 0.14 |  |  |
| ICU LOS | 0.007 | 0.40 |  |  |
| Hospital LOS | 0.003 | 0.55 |  |  |
| MCS | 1.10 (0.51, 2.49) | 0.80 |  |  |
| APACHE II | 0.023 | 0.15 |  |  |
| Mechanical ventilation | 0.92 (0.39, 2.60) | 0.87 |  |  |
| PaO_2_ | 0.067 | 0.01 | -0.032 (-0.088, -0.023) | 0.25 |
| FiO2 | 0.008 | 0.39 |  |  |
| PaO_2_/FiO_2_ ratio | 0.005 | 0.51 |  |  |
| pH | 0.004 | 0.54 |  |  |
| Lactate | 0.006 | 0.49 |  |  |
| Heart rate | 0.016 | 0.22 |  |  |
| MAP | 0.055 | 0.03 | -0.009 (-0.020, -0.002) | 0.10 |
| Creatinine | 0.008 | 0.38 |  |  |
| Urea | 0.001 | 0.91 |  |  |
| Bilirubin | 0.007 | 0.40 |  |  |
| RRT | 0.75 (0.29, 1.76) | 0.53 |  |  |
| Previous CABG | 1.86 (0.28, 8.06) | 0.45 |  |  |
| Heart failure | 0.46 (0.06, 2.19) | 0.40 |  |  |
| Asthma | 1.06 (0.25, 3.42) | 0.94 |  |  |
| COAD | 2.39 (0.32, 11.96) | 0.32 |  |  |
| LVEF | 0.001 | 0.80 |  |  |
| Cardiac index | 0.105 | < 0.01 | -0.001 (-0.001, -3.366) | 0.04 |
| TAPSE | 0.011 | 0.31 |  |  |
| RV S’ | 0.092 | 0.01 | -0.027 (-0.055, -0.018) | 0.31 |
| RVOT VTI | 0.003 | 0.71 |  |  |
| TR Vmax | 0.067 | 0.01 | -0.202 (-0.378, -0.255) | 0.03 |
| RV basal diameter | 0.002 | 0.65 |  |  |
| LA diameter | 0.040 | 0.06 |  |  |
| LA area | 0.003 | 0.32 |  |  |
| RA area | 0.005 | 0.51 |  |  |
| Mitral E velocity | 0.013 | 0.29 |  |  |
| Mitral A velocity | 0.029 | 0.11 |  |  |
| Mitral E/A ratio | 0.003 | 0.58 |  |  |
| DecT | 0.013 | 0.28 |  |  |
| Average E’ | 0.015 | 0.29 |  |  |
| E/E’ | 0.020 | 0.22 |  |  |
| IVC diameter | 0.011 | 0.67 |  |  |

**Supplementary Table 1.** Uni- and Multivariate correlation between clinical and echocardiographic parameters with biplane EBDi. BSA: Body surface area, BMI: Body mass index, CABG: coronary artery bypass grafting, COAD: chronic obstructive airway disease, DecT: E velocity deceleration time. LOS: Length of stay, LVEF: Left ventricular ejection fraction, MAP: Mean arterial pressure, OCHA: Out of hospital cardiac arrest, RRT: Renal replacement therapy, TAPSE: Tricuspid annular plane systolic excursion, RV S’: Tricuspid annulus peak systolic velocity, RVOT VTI: Right ventricular outflow tract velocity time integral.


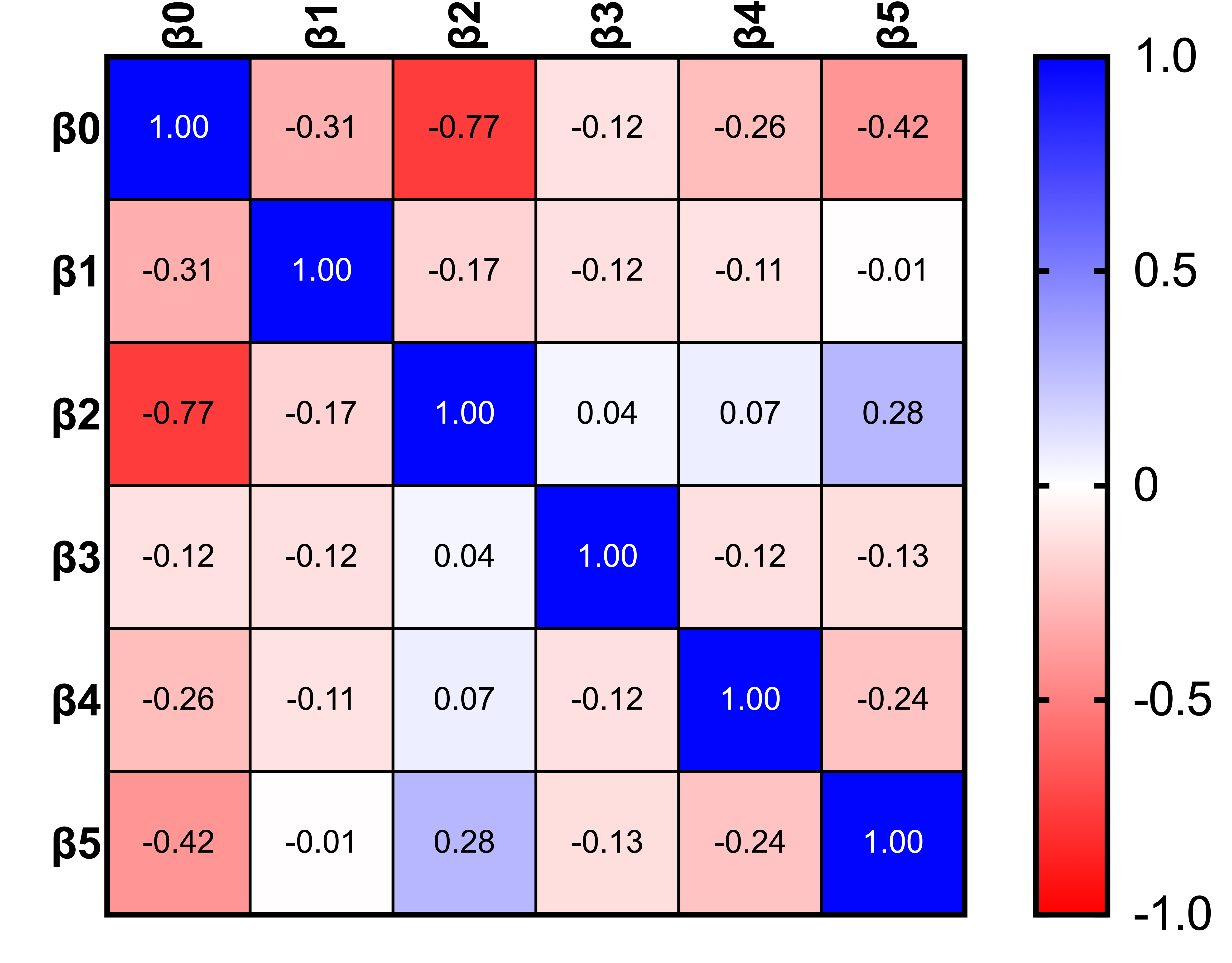


**Supplementary Figure 1**. Co-linearity between the variables included in the multivariate linear regression model. β0: Intercept. β1: PaO_2_. β2: Mean arterial pressure (MAP). β3: Cardiac index (CI). β4: Tricuspid annulus systolic velocity (RV S’). β5: Tricuspid regurgitation maximum velocity (TR Vmax).
